# Estrobolome dysregulation is associated with altered immunometabolism in a mouse model of endometriosis

**DOI:** 10.3389/fendo.2023.1261781

**Published:** 2023-12-08

**Authors:** Hasan Alghetaa, Amira Mohammed, Narendra P. Singh, Ryan F. Bloomquist, Ioulia Chatzistamou, Mitzi Nagarkatti, Prakash Nagarkatti

**Affiliations:** Department of Pathology, Microbiology and Immunology, School of Medicine, University of South Carolina, Columbia, SC, United States

**Keywords:** endometriosis, microbiome, short chain fatty acids, T-cell metabolism, estrobolome, metabolome, immunometabolism

## Abstract

**Introduction:**

Endometriosis is a painful disease that affects around 5% of women of reproductive age. In endometriosis, ectopic endometrial cells or seeded endometrial debris grow in abnormal locations including the peritoneal cavity. Common manifestations of endometriosis include dyspareunia, dysmenorrhea, chronic pelvic pain and often infertility and symptomatic relief or surgical removal are mainstays of treatment. Endometriosis both promotes and responds to estrogen imbalance, leading to intestinal bacterial estrobolome dysregulation and a subsequent induction of inflammation.

**Methods:**

In the current study, we investigated the linkage between gut dysbiosis and immune metabolic response in endometriotic mice. Ovariectomized BALB/c mice received intraperitoneal transplantation of endometrial tissue from OVX donors (OVX+END). Control groups included naïve mice (Naïve), naïve mice that received endometrial transplants (Naive+END) and OVX mice that received the vehicle (OVX+VEH). Colonic content was collected 2 weeks post-transplantation for 16s rRNA pyrosequencing and peritoneal fluid was collected to determine the phenotype of inflammatory cells by flow cytometry.

**Results:**

We noted a significant increase in the number of peritoneal fluid cells, specifically, T cells, natural killer (NK) cells, and NKT cells in OVX+END mice. Phylogenetic taxonomy analysis showed significant dysbiosis in OVX+END mice, with an increase in abundance of Phylum Tenericutes, Class Mollicutes, Order Aneroplasmatales, and Genus Aneroplasma, and a decrease in Order Clostridiales, and Genus Dehalobacterium, when compared to OVX+VEH controls. The metabolomic profile showed an increase in some tricarboxylic acid cycle (TCA)-related metabolites accompanied by a reduction in short-chain fatty acids (SCFA) such as butyric acid in OVX+END mice. Additionally, the mitochondrial and ATP production of immune cells was enforced to a maximal rate in OVX+END mice when compared to OVX+VEH mice.

**Conclusion:**

The current study demonstrates that endometriosis alters the gut microbiota and associated immune metabolism.

## Introduction

Endometriosis is a chronic inflammatory condition and one of the most common gynecological disorders in the world, affecting an estimated 5% of women of childbearing age and resulting in significant global morbidity and medical expenditure (1). In endometriosis, ectopic endometrial glandular and stromal tissues are found outside of the uterus, and, like native endometrium, these tissues respond through growth and proliferation to estrogen-dependent signals (2, 3). Depending on the location of the ectopic tissue, endometriosis can result in significant inflammation, pain, and often infertility. While they can occur almost anywhere, almost all cases of ectopia are found somewhere between the uterine tubes and across the peritoneum, giving rise to the prevailing theory that endometriosis arises through the aberrant retrograde flow of shed endometrial lining up the uterine tubes during the menstrual cycle (4).

The pain and poor response to treatment of endometriosis are commonly attributed to the inflammatory response against ectopic tissue encountered in the disease, particularly during peaks of estrogen release at the transition from the proliferative phase to ovulation during the menstrual cycle (5, 6). Because of the biological role of the endometrium, endometriosis is a strongly estrogen-dependent disorder (7–9) and thus estrogen is considered a mitogen for the inflammatory process (10). Steroid hormones like estrogen are major controllers of reproductive capacity and also serve in the interplay of functions of immunocytes during inflammatory responses (10, 11). As the menstrual cycle progresses from ovulation to the luteal phase, estrogen levels drop and progesterone receptors are upregulated by endometrial tissue. Upon binding of this hormone, proliferation of endometrial tissue slows and moves to a glandular secretory function. While endometriosis tissue has been shown to downregulate progesterone receptors, these hormonal switches in function have been proposed as a mechanism of treatment for endometriosis (2, 11, 12). Other therapies have been proposed or attempted to some degree of success including analgesics for pain management (13), epigenetic regulation (14, 15), hormone therapy (16), dietary supplements (17, 18), surgical removal (13) and other symptomatic treatments (2, 19), but there is still not a reliable therapy for addressing this disorder.

The biological importance of estrogen is not limited to reproductive function, as it also plays a major role in microbiome metabolism (11, 20–22), hematological profile rearrangements (23) as well as in the regulation of immunometabolism (24–26). Intriguingly, while systemic levels of estrogen fluctuate through the normal ovulatory cycle, these steroid fluctuations have not been shown to have a significant effect on the gut microbiota (27). However, during prolonged periods of hormonal up or downregulations, such as during pregnancy, both steroid levels and gut microbiota are altered (28–30). The interplay between gut microbiome and estrogen levels has been designated as the “estrobolome” and is controlled by specific genes. Here, β-glucuronidase enzyme encoded by *gus* gene, native to the gut cleaves conjugated estrogen secreted into the intestine through bile and releases estrogen in its biologically active form (31). Furthermore, *gus* gene is expressed by common gut bacteria including *Ruminococcus gnavus*, *Staphylococcus aureus* and Clostridium (32). The normal estrobolome is essential for regulation of the reproductive cycle and it has been reported that in bacteria-free mice, reproductive capacity is impeded (33) but is then normalized to fertile levels when bacterial recolonization occurs (34). Free estradiol (E2) produced from gut β-glucuronidase together with ovarian produced E2 acts in concert to stimulate the immune system in a cyclical manner (24). During these immunological responses, macrophages increase their rate of glycolytic and tricarboxylic acid cycle shortly after being activated (35) and effector T cells accommodate glycolytic pathways in production of ATP molecules (36).

In this study we elucidated the dysregulation of immunometabolism as a response to estrobolome alterations during endometriosis. By seeding stimulated endometrial tissue to the peritoneum in syngeneic mouse transplant experiments, we explored the resulting perturbations of systemic inflammatory cells, gut microbiome, metabolome and immunometabolism encountered in endometriosis to obtain holistic understanding of the nature of changes occurring during this clinical disorder.

## Materials and methods

### Experimental animals

Female BALB/c mice aged between 6 – 8 weeks utilized in this study were purchased from The Jackson Laboratory and acclimatized for at least one week after delivery. Randomized grouping of animals was performed and every 4 mice were housed in same cage till the end of experiments. All study mice were exposed to same pathogen-free housing conditions with freely accessible food (standard chow) and water, 18 – 25°C temperature, and alternating 12 light/12 dark. Approval from the Institutional Animal Care and Use Committee (IUCAC) and from the University of South Carolina was obtained before performance of any study experiments (AUP2374).

### Materials

Surgical instruments including Castroviejo scissors, uniband LA-1 micro point scissors, serrated jaw MF-2 micro forceps, fully curved micro forceps MF-3, straight micro forceps soldering tweezers, insertion/extraction tweezers and anti-wicking tweezers were purchased from Cedarlane labs – Canada. Surgical sutures including 5-0 Perma Hand Silk Black 1X18’’ PS-3, 5-0 Perma Hand Silk Black 2X60’’ no needle and 4-0 Perma Hand Silk Black 1X18’’ G-3 were purchased from the Ethicon – USA. Diethylstilbestrol (DES) was purchased from Sigma – USA and prepared in mineral oil (Sigma-Aldrich, USA). GIMA 2mm diameter biopsy punch (GIMA, UK) was used to divide the endometrial layer into 2mm pieces. An 18G syringe needle (BD, USA) attached to a tuberculin syringe (BD, USA) was used to deliver endometrial specimens into the peritoneal cavity. Banamine (Merck, USA) solution was used every 12 hours for 2 two days to alleviate the pain during post-surgical care period. DMEM/F12 medium was used for preparation of endometrial transplant tissue (Sigma-Aldrich, USA). Skin incisions were closed with a Reflex Clip Applier (World Precision Instrument, USA) and clips were removed using Reflex Clip Removing Forceps (World Precision Instrument, USA).

### Induction of experimental endometriosis

As the endometriosis is an estrogen-dependent disorder, mouse model was designed to mimic this disorder by using ovariectomized mice treated with estradiol (37). Briefly, donor and recipient mice were anaesthetized with inhaled isoflurane before being surgically prepared. The right and left ovaries with attached salpinx were ligated and removed. Muscular layers were sutured with silk, while the skin was closed with metal clips. Systemic analgesia, Benamine, was given every 12 hours post-surgery for 48 hours. Seven days later, the clips were removed and OVX mice were used.

**Figure 1:**
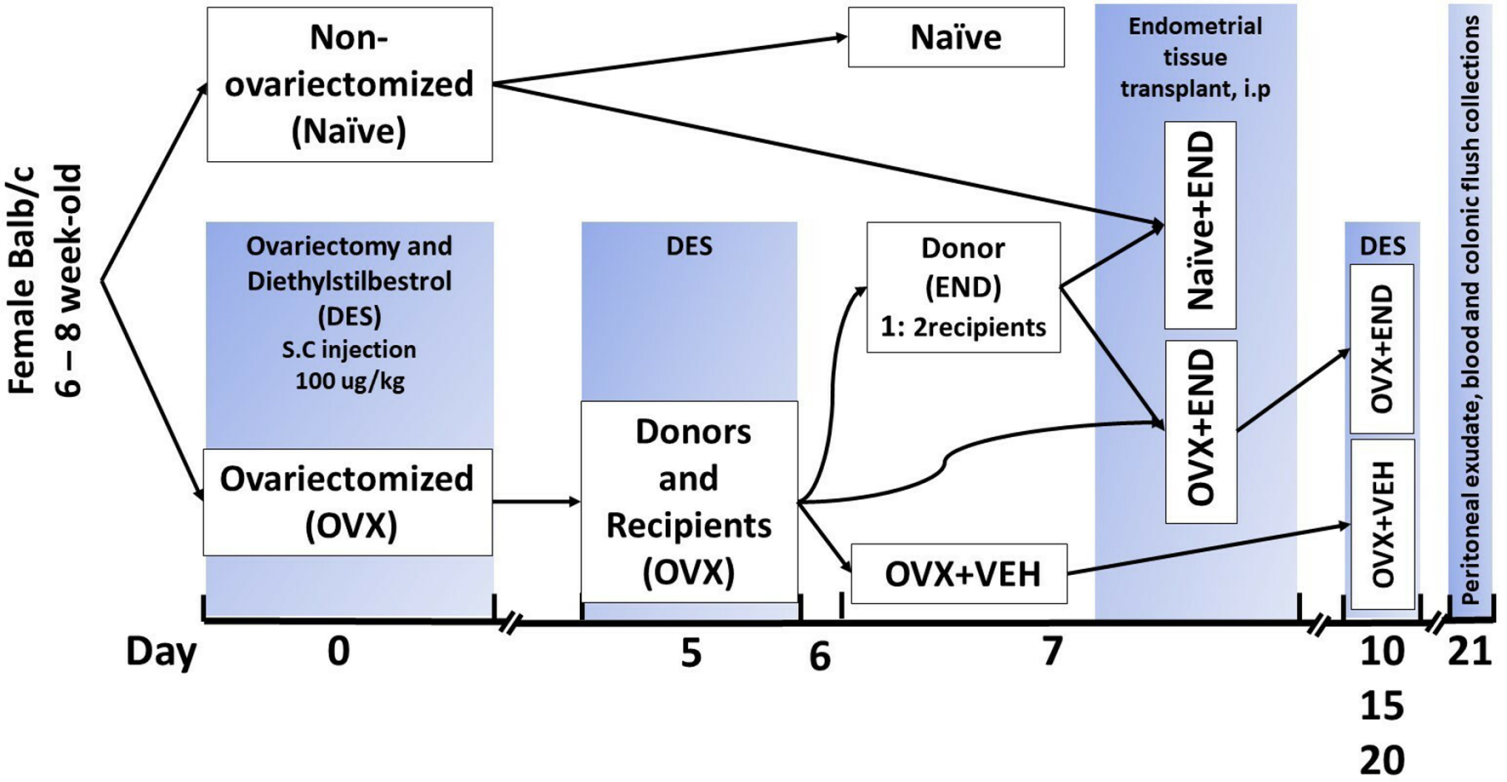
Experimental design. Mice were ovariectomized (OVX) and received analgesia after finishing the surgery and then repeated every 12 hours during post-surgical care period up to 48 hours to alleviate the pain. All ovariectomized mice were injected subcutaneously with diethylstilbesterol (DES) for 5 days after removal of ovaries and then repeated every 5 days till end of experiment. Closing clips were removed from all ovariectomized mice at day 7 post-surgical operation. Ovariectomized mice were divided into donors and recipients on day 7 at a ratio of 1:2. Harvested endometrial tissues (END) from OVX mice were transplanted into OVX (OVX+END) or non-ovariectomized (Naïve+END) recipient mice. As controls, we used non-ovariectomized naïve mice (Naïve), Naïve mice that received endometrial tissue (Naïve+END), and ovariectomized mice injected with vehicle (OVX+VEH).

The endometrial tissue was used to induce the experimental endometriosis in mice as described (38, 39). The schematic details are also provided in **Figure 1**. Briefly, BALB/c female mice were assigned to one of four experimental groups (**Figure 1**): OVX+END, ovariectomized mice receiving transplanted endometrial tissue; OVX+VEH, ovariectomized mice injected intraperitoneally with PBS but without transplant; Naïve (N) mice, non-ovariectomized mice with no treatment as control group and lastly, Naïve+END (NE), non-ovariectomized mice receiving transplanted endometrial tissue. All DES treatments were performed as 100µg/kg of DES injected subcutaneously. In the OVX+END and OVX+VEH groups (collectively considered ovariectomized-OVX), a dose of DES was delivered and ovariectomy was performed at day 0, while N and NE groups were housed without intervention. On day 5, OVX mice received another injection of DES in order to stimulate endometrial growth while N and NE groups were still under the influence of endogenous estrogen from their ovaries. Here, N group received no further intervention until they were euthanized. On day 7, OVX mice were split into OVX+VEH group, endometrium transplant donor group (END), and the OVX+END group described above to receive the transplanted tissue. One donor endometrium (END) provided enough tissue to seed the peritoneum of two recipient mice.

The donor mice were euthanized via overdose of isoflurane inhaled anesthesia. In a petri dish containing DMEM/F12 medium, the uterine horns of donor mice were longitudinally opened, and the endometrial layer was pealed from the myometrium and serosa layers using micro forceps and then divided into approximately 2mm pieces. This tissue was then transferred to another petri dish containing phosphate buffer saline (PBS). Approximately 35µg (wet weight) of the endometrial tissue samples were loaded into a 1ml syringe via an 18g needle in a total volume of 500 µl PBS. Loaded endometrial tissues were then transplanted to the peritoneal cavity through abdominal wall of either OVX+END or Naïve+END recipients to complete the transplant procedure.

At days 10, 15, and 20, the OVX+END and OVX+VEH groups received injections of DES, post ovariectomy procedure, while Naïve+END and Naïve did not receive DES still under hormonal influence of intact ovaries. The experimental endpoint was 14 days post endometrial transplant, which was 21 days post OVX procedure.

### Tissue samplings for downstream analysis

At the endpoint of the experiments (14 days post-transplantation experiments), all study mice were anesthetized and euthanized under isoflurane for tissue sampling and downstream analysis. Blood samples were collected for complete blood count by VetScan analyzer system (Abaxis, USA) and for serological analysis. Single cell suspension of peritoneal fluid (PF) was prepared by using RBCs-lysis buffer (Sigma-Aldrich, USA) and strained with the 70-micron strainer (ThermoFisher, USA) to be utilized in either real-time metabolism analyzer Seahorse (Agilent, USA) or FACS-Celesta flowcytometry sorting system (BD, USA). Colon flushes (CF) were collected from the colon under aseptic conditions in sterile PBS for microbiome analysis. CF for metabolomic and short chain fatty acids analysis were collected in sterile distilled water. Uterine horns were excised for histopathological investigation with hematoxylin and eosin (H&E) staining and to collect uterine horn lavage fluid (ULF).

### Inflammatory cell counting of peritoneal fluid and uterine lavage fluid

PF was collected by injecting the mice under deep anesthesia by isoflurane, then the skin of abdominal wall was opened to expose the peritoneal sac. Five milliliters of sterile PBS were intraperitoneally injected and the mouse then rolled thoroughly for 3 – 5 minutes to elute all the peritoneal traces and cells. ULF was collected by slow-passing 2 – 3 ml of PBS and collected in centrifuge tubes. Collected PF and ULF was then transferred to conical tubes for centrifugation and separation of the supernatant and stored at -80°C for analysis. While the pelleted cells were treated with RBC lysis buffer for 30 – 60 seconds and then blocked by using cold 10%-FBS buffer and washed with cold FACS for cell counting. Cells were resuspended in FACS buffer for counting by using Trypan blue staining and TC20 automated cell counter (40).

### Mononuclear cell isolation and T cells subset determination from peritoneal fluid

Briefly, peritoneal fluid was collected in cold FACS and processed into single cell suspension by using RBC lysis buffer (Sigma-Aldrich, USA) for about 60 seconds and then washed with cold FACS. Then all samples were filtered with 70µm strainer (ThermoFisher, USA), centrifuged at 4°C, 1000 RPM for 10 minutes and then the cell pellets were resuspended in cold FACS. To determine the T cell and natural killer (NK) subset, isolated cells were stained with anti-CD3-APC and NK1.1-BB515 antibodies (Biolegend, USA), respectively. Finally, the stained samples were analyzed by using BD-FACS Celesta flow cytometry system (BD, USA) and acquired data were visualized by using built-in Diva software (BD, USA) as described before (41).

### Peripheral blood count assessment

Whole blood was obtained via retroorbital vein rupture by using heparinized capillary tubes inserted into medial canthus of isoflurane-anesthetized mice. The collected blood samples were kept in heparinized tube before being transferred to be analyzed by blood-autoanalyzer system, Vetscan HM5 hematology analyzer (Abaxis, USA) for complete blood count assessment.

### ^3^H-Thymidine incorporation assay

To evaluate the proliferative capacity of PF inflammatory cells, 10^5^ cells per well were seeded in DMEM/F12 medium for 12 hours incubated with 1µCui/well of ^3^H-thymidine isotope at 37°C and 5% CO2, then radioactivity was measured using MicroBeta Trilux liquid-scintillation counter to (36, 42).

### Histopathological examination of uterine tissue

Harvested uterine horns were fixed in 4% paraformaldehyde overnight and then stored in PBS at 4°C till the time of sectioning and staining with hematoxylin and eosin (43).

### Colonic microbiota analysis

The collected colonic flushes were prepared for 16s rRNA as described by previous publications (44, 45).

### Measurement of short chain fatty acids levels in colonic flushes

Briefly, colons of study mice were opened and about 100 mg of luminal solid contents were removed and then suspended with distilled water. Collected flushes were centrifuged to separate the supernatant and stored at -80°C for future use. At time of SCFA analysis, all samples were thawed gradually in ice box and then acidified by hydrochloric acid (HCL) before adding 4-methylvaleric acid as internal standard. Gas chromograph CP-3800 (Varian) and spectrometry mass (GC-MS) system was used to quantify the SCFAs. Varian MS Workstation (version 6.9.2) was used to collect and analyze acquired data. Finally, the linear equation was used to calculate the concentrations of SCFAs in each sample (41, 46).

### Glycolytic and tricarboxylic acid metabolites measurement and metabolome profiling

Serum was separated from study mice and used for metabolic extraction by using liquid chromatograph mass spectrometry (LC-MS) as described in our previous study (36). Briefly, metabolites derived from the TCA and glycolysis were characterized by using 5mM of ammonium acetate (pH9.9) and other buffers in assistance of Luna 3µM NH2 100 A° chromatography column (Phenomenex, CA). All identified metabolites were normalized to internal standard (47).

### Real-time metabolism analysis of mitochondrial respiration by XFp Seahorse analyzer

These two metabolic pathways were evaluated by using real-time metabolism analyzer Seahorse (Agilent, USA) according to the manufacturer’s protocols. The principle of measurements was based on the calculation of oxygen consumption rate (OCR) and extra cellular acidification rate (ECAR). Cells (3X10^5^) taken from single cell suspension of PF was seeded into XFp miniplates by assistance of Cell-Tak biological adhesive (Corning, USA). At the end of analyzer run, Hoechst 33342 dye (Invitrogen, USA) was applied to the plate wells to count the remaining live cells in order to normalize the acquired data based on the number of living cells used in this run. Counting of live cells was performed by using automated imaging microscope, Cytation5 Imaging System (BioTek, USA). Generated data by Seahorse were transferred to be interpreted by Seahorse Wave Desktop Software (Agilent, USA) to calculate the kinetic energy (36).

### Statistical analysis

All experiments of this study were repeated 3 independent times. Sample sizes varied between 3 – 5 mice per group and the numbers are stated in the figure legend. Metabolomes were normalized and analyzed as Log2 examined by t-test by using R-studio (R Studio Inc, USA) as described previously (36). One-Way ANOVA test was applied whenever there were more than two groups to compare with multiple Tukey’s correction. The t-test was applied with Holm-Sidak correction method to compare two groups. p<0.05 considered as significant threshold and the levels were depicted as: *p<0.05, **p<0.01, ***p<0.001 and ^#^p<0.0001. When statistical comparison includes more than three groups, different lowercase letters were used to depict significant differences among these groups. For example, groups having the same letters ‘a’ would be insignificant while groups depicting ‘a’ and ‘b’ would be statistically significant.

## Results

### Endometrial transplants in OVX mice trigger an exacerbated inflammatory response in the peritoneal cavity and peripheral blood

**Figure 2:**
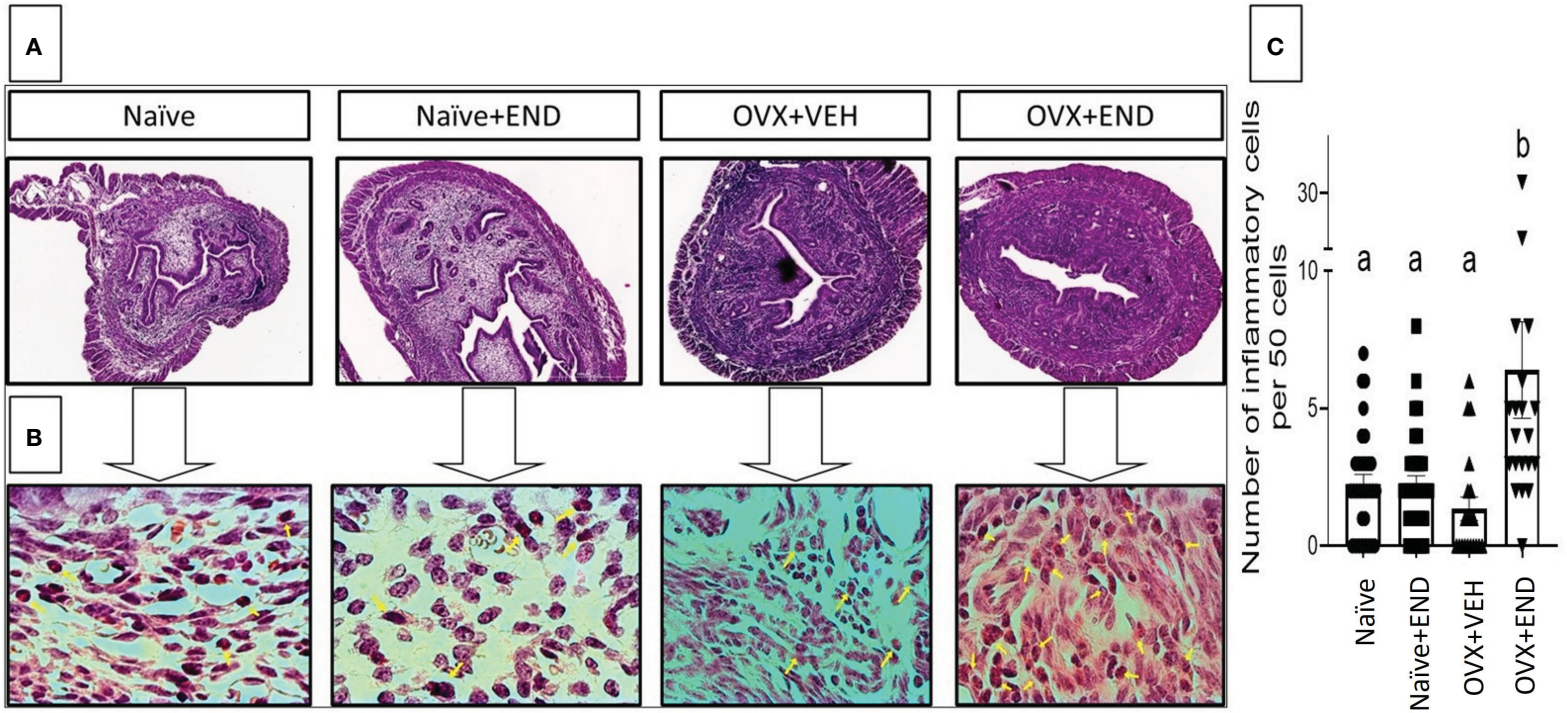
Histopathological investigation of uterine horn 14 days post-endometrial tissue transplantation. H&E-stained cross-sections of uterine horn of different experimental groups, (n=4) examined under **(A)** 4X and **(B)** 100X; yellow arrows point the inflammatory cells in the uterine parenchyma. **(C)** Statistical analysis of counted inflammatory cells per 50 cells in every examined field (n= 20–31) calculated by One-way ANOVA test. Different lowercase letters depict significant differences when p<0.05. For detailed P-value, please check **Supplementary Table S1**.

**Figure 3:**
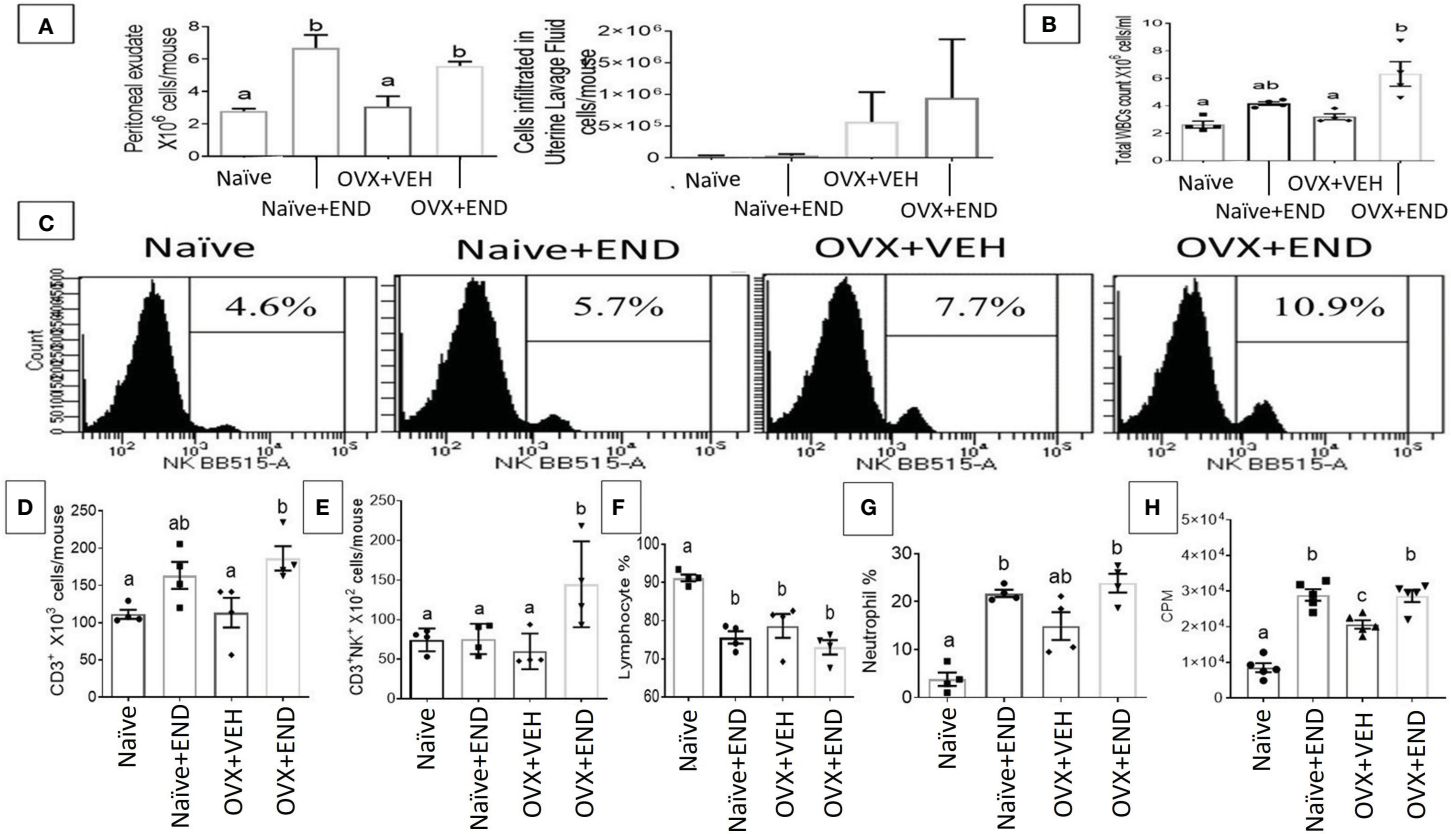
Evaluation of endometriosis induction 14 days post-endometrial tissue transplantation. **(A)** Total number of inflammatory cells in the peritoneal cavity (left) and uterine lavage fluid (right) counted by using hemocytometer chamber with trypan blue dye. **(B)** Total white blood cells in peripheral blood measured by Vetscan HM5, (n=4). **(C)** Flow cytometry results of peritoneal fluid (PF) cells stained for natural killer (NK) population, (n=4). **(D)** Total number of CD3+ T-lymphocytes in PF, (n=4). **(E)** Total natural killer T-cells (CD3^+^NK^+^) in PF, (n=4). **(F)** Percentage of lymphocyte population in the peripheral blood, (n=4). **(G)** Percentage of neutrophil population in the peripheral blood, (n=4). **(H)** Proliferation of inflammatory cells in the peritoneal cavity isolated from different study groups detected using ^3^H-thymidine-incorporation assay, (n=5). One-way ANOVA statistical analysis used for panels **(B, D-H)**. Different lowercase letters depict significant differences when p<0.05. CPM, Counts per minute, For detailed P-value, please check **Supplementary Table S2**.

At fourteen days post-receiving endometrial transplantation, local and systemic immunologic responses were evident (**Figures 2**, **3**). Histopathological investigation of uterine horns excised from the four groups of mice using H&E staining showed that in the OVX+END group there was an increase in the inflammatory cells (yellow arrows) to the endometrium as well as myometrium (**Figures 2A, B**) when compared to the control OVX+Veh or Naive groups. By using ImageJ software, the statistical analysis showed significant increase in the number of inflammatory cells (mostly neutrophils) in the endometrium (endometritis) as well as in myometrium (myometritis) of OVX+END group in comparison with all other study groups (**Figure 2C**). There was also significant augmentation (p<0.05) in the inflammatory cells in the peritoneal cavity of OVX+END group when compared to OVX+VEH or Naïve groups (**Figure 3A**, left panel). Interestingly, the Naïve+END group also behaved similar to OVX+END group. The cell counts in the uterine lumen were not significantly altered in all groups tested (**Figure 2A**, right panel). The total WBC counts in the peripheral blood of the OVX+END group was significantly higher (p<0.05) than the OVX+VEH and naïve mice (**Figure 3B**, right panel). The percentage of NK cells were higher in the OVX+END group when compared to other groups (**Figure 3C**). The total number of CD3^+^ T cells and CD3^+^NK^+^ cells in the peritoneal cavity were found to be significantly increased (p<0.05) in the OVX+END group when compared to Naïve or OVX+VEH groups (**Figures 3D, E**). The percentage of lymphocytes and neutrophils in the peripheral blood were studied too. When we tested the percentage of lymphocytes in the blood, it was decreased in all groups when compared to the naïve group (**Figure 3F**). The neutrophil percentage in the peripheral blood of mice transplanted with endometrial tissue (Naïve+END, OVX+END) was significantly higher (P<0.05) than in the non-transplanted mice (**Figure 3G**). ^3^H-thymidine incorporation assay was performed to estimate the proliferative capacity of the inflammatory cells in the peritoneal fluid and to assess the ability of these cells to proliferate. Interestingly, the results showed that there was significant increase (p<0.05) in proliferation capacity of inflammatory cells isolated from OVX+END group when compared to Naïve or OVX+VEH group. The Naïve+END mice also demonstrated a similar response as the OVX+END mice (**Figure 3H**). Overall, the results indicated that the endometrial transplants in OVX mice were associated with an exacerbated inflammatory response in the peritoneal cavity and peripheral blood. It was interesting to note that naïve mice that received the endometrial transplant (Naïve+END), also exhibited some immunological changes like OVX+END group such as increase in the percentages of neutrophils while with respect to most of the immunological profiles studied, they behaved like the naïve controls.

### Endometrial transplantation in Naïve or OVX mice leads to enhanced gus-enriched bacteria like Ruminococcus spp

**Figure 4:**
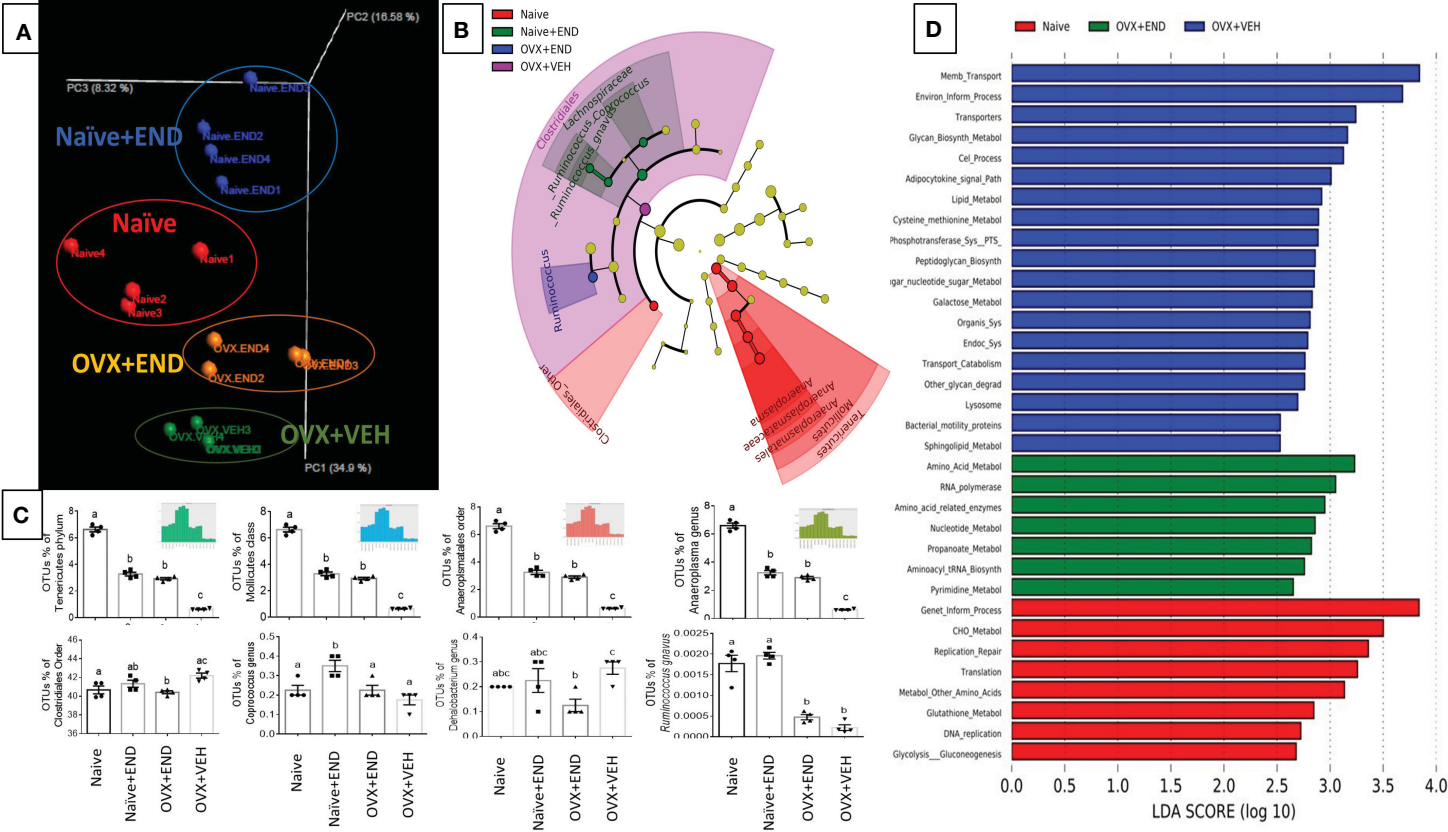
Characterization of bacterial communities and their functional metabolism pathways via 16s rRNA metagenomic analysis. **(A)** Unweighted beta-diversity of the commensal gut microbiome. **(B)** Cladogram using results of the linear discriminant analysis (LDA) model of bacterial hierarchy. **(C)** One-way ANOVA statistical comparisons among the experimental groups based on the percentage of occupational taxonomy units (OTUs) of biomarker bacteria. **(D)** LDA analysis of the microbial metabolic pathways via metagenomic functional prediction of Phylogenetic Investigation of Communities by Reconstruction of Unobserved States (PICRUST). Different lowercase letters in **(C)** indicates significant differences when p<0.05. (n=4). For detailed P-value, please check **Supplementary Table S3**.

Investigation of gut microbial communities via sequencing the 16s rRNA V3-V4 regions showed that there was dysbiosis in the microbial environment of the colon in all experimental groups compared to the Naïve group (**Figure 4**). Bioinformatic analysis of our collected data showed that the commensal bacteria of individual mice were clustered closely within their own group, while the 4 groups tested showed clear segregation (**Figure 4A**). A cladogram generated from linear discriminant analysis effect size (LEfSe) demonstrated that Clostridiales_order, Tenericutes phylum and its down tree ancestry, Mollicutes class, Anaerplasmatales order, Anaeroplasmaceae family, and Anaeroplasma genus constituted the detectable commensal bacteria in the guts of the Naïve group (**Figure 4B**). The Naïve+END and OVX+END mice showed simultaneous deviation from Naive and OVX+VEH gut microbiotas and showed similarities between each other with the enrichment of Phylum Tenericutes, Class Mollicutes, Order Aneroplasmatales, and Genus Aneroplasma. Lacnospiraceae family and its Coprococcus genus, Ruminococcus genus, and *Ruminococcus gnavus* were commensal biomarkers in the Naïve+END group that were not present in the Naïve group (**Figure 4B**). Also, Clostridiales order was the biomarker bacteria in the colons of OVX+VEH (**Figure 4B**). Statistical analysis of occupational taxonomy units (OTUs) of bacteria showed significant alterations (p<0.05) among the study groups (**Figure 4C**). Furthermore, linear discriminant analysis (LDA) scores set at >2-fold change among the study groups (Naïve, Naive +END, OVX+VEH and OVX+END), using PICRUST-generated level 3 KEGG pathways of 16s rRNA data revealed that there was no biomarker functional pathway of the bacteria for Naïve+END group (**Figure 4D**). The other groups showed different pathways involved in the bacterial metabolism that are related to the host metabolism (**Figure 4D**). Together, these data suggested that each treatment group had a distinct population of bacteria (**Figure 4A**) and that Naïve or OVX mice that received endometrial transplants exhibited enhanced *gus*-enriched bacteria like Ruminococcus spp. which in turn could improve the E2 metabolism as a consequence (**Figure 4B**).

### Short-chain fatty acids produced by gut microbiota exhibit differential expression following endometrial transplants

**Figure 5:**
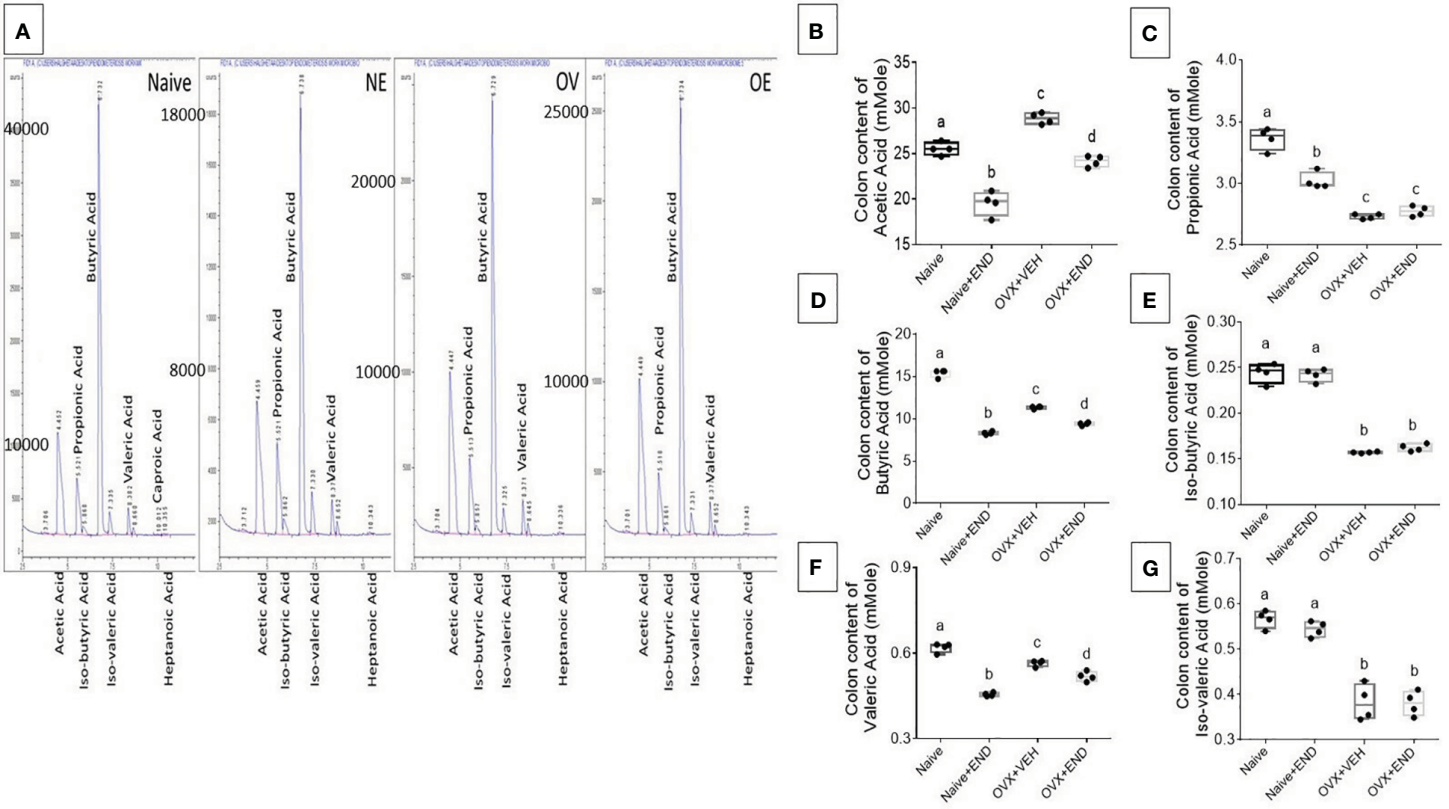
Colonic enrichment of short chain fatty acids (SCFAs). **(A)** GC-MS panels showing peaks of SCFAs. **(B–G)** One-way ANOVA statistical analysis of the colonic content of SCFAs, (n=4). **(B)** Acetic acid. **(C)** Propionic acid. **(D)** Butyric acid. **(E)** Iso-butyric acid. **(F)** Valeric acid. **(G)** Iso-Valeric acid. Different lowercase letters in **(C)** indicates significant differences when p<0.05. For detailed P-value, please check **Supplementary Table S4**.

Next, we studied the bacterial end-product concentrations in the guts represented by the short chain fatty acids (SCFAs) by using Gas Chromatography-Mass Spectrometry (GC-MS) showed in **Figure 5A**. Upon examination of the 4 groups of mice, the overall trend indicated that significant (p<0.05) decreased levels of acetic acid (**Figure 5B**), propionic acid (**Figure 5C**), butyric acid (**Figure 5D**) and valeric acid (**Figure 5E**) in OVX+END group when compared to the Naïve group. Similar changes were also seen in OVX+VEH group suggesting that ovariectomy and DES treatment had significant impact on colonic SCFAs. The Naïve+END group also exhibited both similarities and differences in SCFAs when compared to the Naïve group thereby suggesting that endometrial transplants in naïve mice also induced some changes in SCFAs. Together, these data suggested that bacterial metabolites, SCFAs, exhibit differential expression following endometrial transplants and because SCFAs also regulate immune cells, the SCFAs may play a role in immune response during endometriosis.

### Endometrial transplantation enhances the immunometabolism in inflammatory cells and altered plasma metabolome

**Figure 6:**
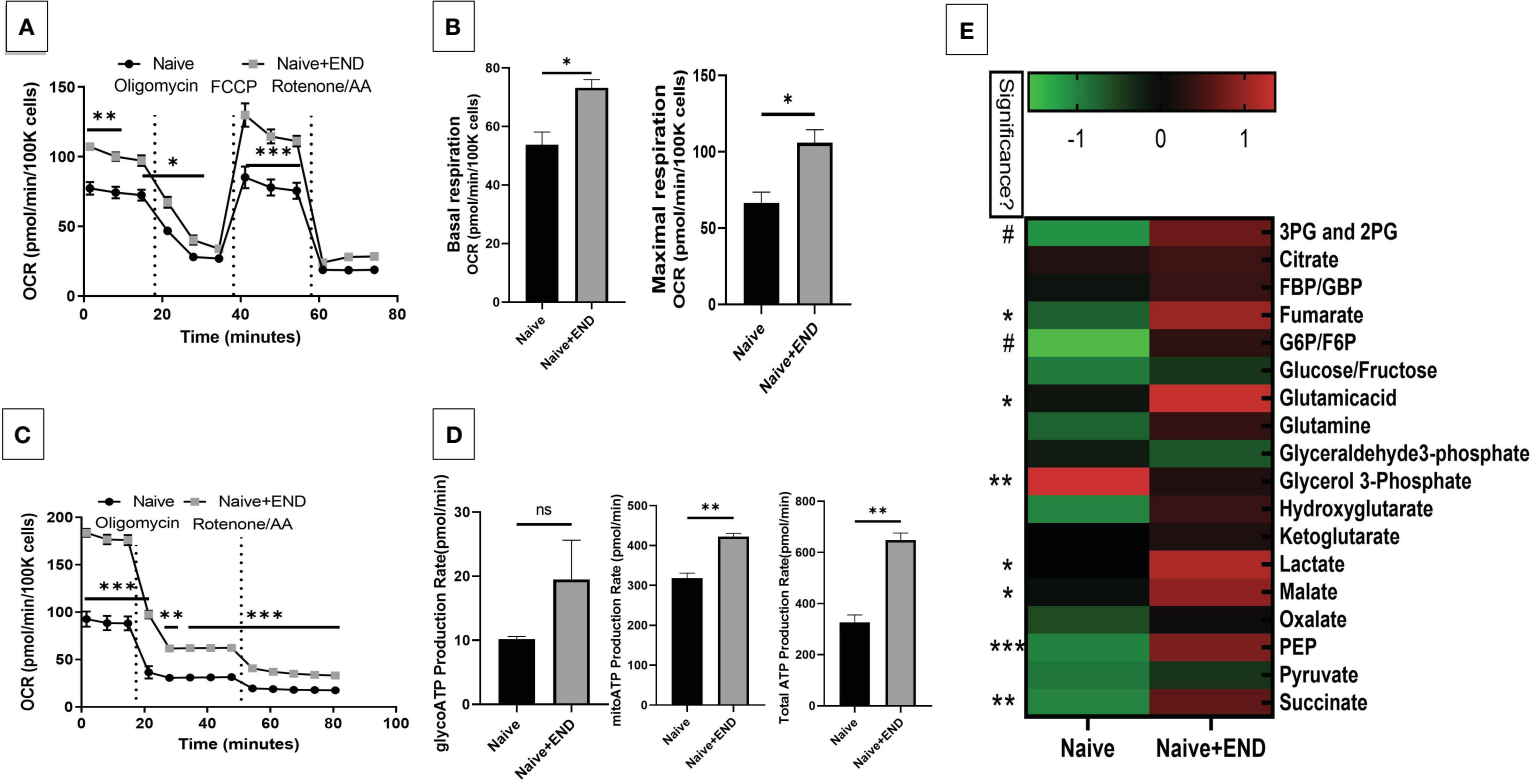
Effect of metabolic pathways of PF inflammatory cells in non-ovariectomized mice. **(A)** Cell Mito Stress test detected by real-time metabolism analyzer Seahorse (n=3). FCCP: carbonyl cyanide p-(trifluoromethoxy)phenylhydrazone. AA: antimycin **(A. B)** Kinetic calculation of basal (left) and maximal (right) mitochondrial respiration. **(C)** ATP production rate test detected by real-time metabolism analyzer Seahorse (n=3). **(D)** Kinetic calculations of glycolytic ATP production rate (left), mitochondrial ATP production rate (middle) and total ATP production rate (right). **(E)** LC-MS showing the plasma metabolomic profile regarding TCA and glycolysis pathways, (n=4). ns, not significant; *p<0.05, **p<0.01, ***p<0.001, #p<0.0001.

**Figure 7:**
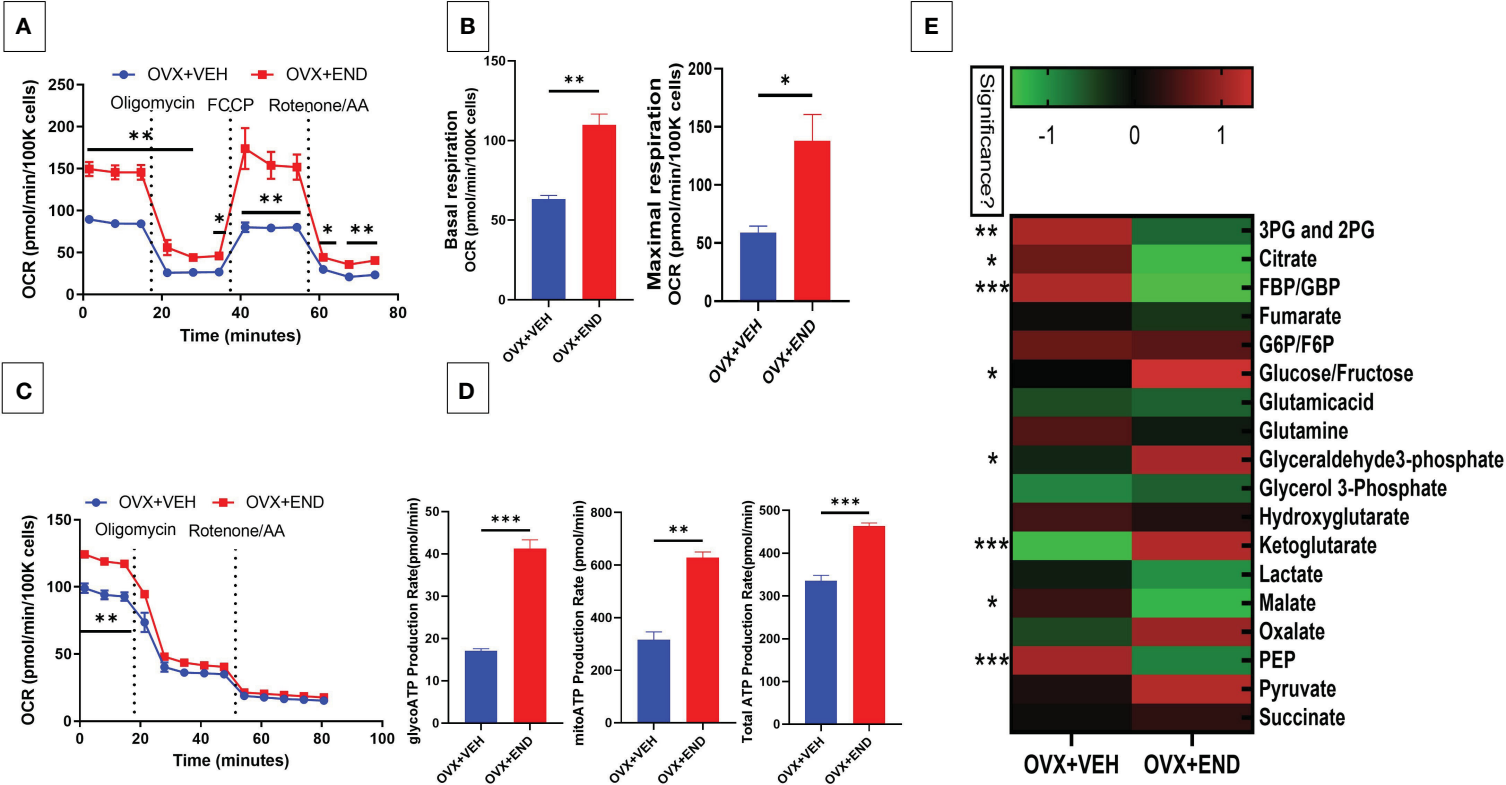
Effect of metabolic pathways of PF inflammatory cells in ovariectomized mice. **(A)** Cell Mito Stress test detected by real-time metabolism analyzer Seahorse (n=3). FCCP: carbonyl cyanide p-(trifluoromethoxy)phenylhydrazone. AA: antimycin **(A. B)** Kinetic calculation of basal (left) and maximal (right) mitochondrial respiration. **(C)** ATP production rate test detected by real-time metabolism analyzer Seahorse (n=3). **(D)** Kinetic calculations of glycolytic ATP production rate (left), mitochondrial ATP production rate (middle) and total ATP production rate (right). **(E)** LC-MS showing the plasma metabolomic profile regarding the TCA and glycolysis pathways, (n=4). *p<0.05, **p0.01, ***p<0.001.

We next studied the interplay between estrobolome dysregulation of microbiota and their potential influence on the immune cells’ metabolomic response in the endometriosis model using cells from the peritoneal cavity. These cells were seeded into specific culture plates of XFp Seahorse real-time metabolism analyzer before being incubated and analyzed based on calculation of oxygen consumption rate and extracellular acidification rate to estimate the ATP production rate (**Figures 6**, **7**). Real-time metabolism analysis revealed that the basal and stressed mitochondrial respiration of immune cells were significantly (p<0.05) higher in the Naïve+END (**Figures 6A, B**) and OVX+END groups (**Figures 7A, B**) in comparison to non-transplanted mice of Naïve and OVX+VEH groups, respectively. Furthermore, the main source of ATP production in the Naïve group was via mitochondrial respiration (**Figures 6C, D**). However, transplantation of endometrial tissues led to significantly (p<0.05) accelerated mitochondrial ATP production which in turn significantly (p<0.05) increased the total ATP molecules in cells from Naïve+END group (**Figure 6D**). In the OVX groups, endometrial transplantation modified the respiratory pathways of inflammatory cells (**Figures 7B, C**). Stimulating the inflammatory cells in the peritoneal cavity of OVX+END mice led to further significant (p<0.05) increase in the rate of ATP production via mitochondrial and glycolytic processes in comparison with OVX-VEH group (**Figure 7D**). Together, these data suggested that endometrial transplantation-induced immune cells exhibit accelerated metabolic rate.

TCA-related metabolites levels, such as fumarate, glutamate, malate and succinate were significantly (p<0.05) higher in Naïve+END group than in Naïve mice (**Figure 6E**). Interestingly, glycolysis-related metabolites such as 3-phosphoglycerate and 2-phosphglycerate (3PG and 2PG), glucose-6-phosphate and fructose-6-phosphate (G6P/F6P) and phosphoenolpyruvate (PEP) were significantly (p<0.05) elevated in the plasma of Naïve+END mice in comparison to Naïve group (**Figure 6E**). Interestingly, when such metabolites were studied in OVX+END mice, some of the metabolites showed opposite results. For example, the levels of 3PG, 2PG, lactate, malate, PEP were decreased while some such as glucose/fructose, glyceraldehyde, ketoglutarate, oxalate, and pyruvate were increased in OVX+END when compared to the OVX+VEH group (**Figure 7E**). It was noteworthy that several of the metabolite expression profile was different between OVX+END vs Naïve+END group (**Figure 6E** vs **Figure 7E**), thereby suggesting that ovariectomy did affect the metabolomic profile in addition to endometrial transplant.

## Discussion

For decades, researchers have investigated the interface between the metabolism and immune response in various physiological and pathological conditions, such as inflammatory bowel diseases (48), cardiovascular disease (49), and in a myriad of cellular models (36, 41, 50–55). The development of endometriosis is regulated by estrogen metabolism and host inflammation, which in turn are influenced by several factors such as the microbiome and the estrobolome (56). However, to date, few estrobolome studies have been performed to investigate the estrogen-microbiome axis across disease (20, 57, 58). While the study of the relationship between the estrobolome-endometriosis-gut microbiome-axis is ripe for discovery, the multifactorial causes of endometriosis add challenges to understanding its pathogenesis (12).

Many studies have been performed that associate endometriosis with changes in gut microbiome (59). It is also well established that endometriosis is a chronic estrogen-dependent inflammatory disease with endometrial stroma and glands outside the uterine cavity (60). However, whether there is cross-talk between gut dysbiosis and inflammation is not clear. Moreover, how the metabolites produced by the endometriosis-induced gut microbiota alter the immune metabolism in the inflammatory cells has not been previously studied. In the current study, we tried to connect all such events induced during endometriosis by using both naïve and OVX mice to get a better understanding of the cross-talk between endometriosis, gut microbiota and immune functions in the host.

There are several murine models of endometriosis (61). In the present study, we used the well-established mouse model of endometriosis in which the endometrial tissue is injected intraperitoneally into the recipient OVX mice (39). Such models have also been compared to models in which the recipient mice are intact that have not undergone ovariectomy (62), similar to our Naïve+END group. Both models develop endometrial lesions although the progression of the disease varies to some extent. In the current study, we noted that OVX+END group demonstrated similarities to the Naïve+END group with respect to the induction of some changes in the microbiota and immune profile while differing in some respects. For example, both OVX+END and Naïve+END groups showed similar induction of peritoneal inflammatory cells and neutrophils in the blood. Also, both groups exhibited increased presence of Ruminococcus genus. However, the OVX+END and Naïve+END groups showed significant difference in their expression of certain microbes, SCFA, and metabolite profile. The main difference between OVX+END and Naïve+END mice is that the former lacked the ovarian hormones but received exogenous administration of estrogen, while the Naïve+END mice had intact hypothalamic-pituitary-ovarian axis which controls several hormones and regulate female reproduction. Thus, our studies suggested that such hormones may play a role in regulating the microbial dysbiosis and immune metabolism during endometriosis.

In the present study, we investigated the microbial dysbiosis and the immunocyte metabolism triggered following endometrial transplants. Endometriosis is best hypothesized to arise from abnormal growth of endometrium-like tissue derived from escaped uterus that get implanted in the pelvic and abdominal cavity wall (13). This triggers an inflammatory response consistent with our observation of an excessive number of immune cells in the parenchyma of uterus (**Figures 2A-C**), peritoneal fluid, and an increase in total WBC counts in peripheral blood (**Figures 3A-G**). Accumulations of such cells is hypothesized to contribute to the decline in fertility rate and pain with menstruation in endometriosis (2, 13, 19). The immune cells that infiltrate is heterogeneous. Fukui et al. showed the infiltration of NK cells to the peritoneal cavity leading to inflammation during endometriosis (63). In addition, plasma cells in the uterine tissue could also serve as a biomarker of non-invasive diagnosis of endometriosis (3). Other cells reported to infiltrate include dendritic cells and neutrophils (3, 5, 64). In the current study, we noted an increase in CD3^+^T cells, NK cells, and NKT cells in the uterine cavity as well as increases in lymphocytes and neutrophils in the blood.

The aggregation of different immunocytes in the peritoneum seen in endometriosis has the potential to increase levels of reactive oxygen species present, which in turn could enhance inflammatory cell proliferation (**Figure 3H**) and leading to tissue damage (6). Clinical trials have been performed to treat endometriosis via suppression of the inflammatory response from oxidative stress using herbal-derivative anti-oxidants (13, 17, 18), miRNA regulation (14), or hormonal manipulation (11, 14). It has been hypothesized that there is an integral interplay between the gastrointestinal microbiome and estrogen and other hormones (16, 22, 31). We therefore examined perturbations of the gut-microbiota to determine if endometrial transplantation altered the gastrointestinal homeostasis and microbiome profile (**Figure 4**). Interestingly, our results showed that there was a distinct separation between the microbial communities of each study group (**Figure 4A**). Moreover, we found that there was a biomarker bacteria for each group of Naïve (Tenericutes, Mollicutes, Anaeroplasmatales, Anaeroplasmataceae and Anaeroplasma), Naïve+END (Lachnospiraceae family includes Coprocuccus and [Ruminococcus] genera), OVX+VEH (Clostrediales order), and OVX+END (*Ruminococcus* spp) (**Figure 4B**). Interestingly, most of biomarker bacteria in all study groups except Naïve mice shared one gene involvement, the *gus*-gene, that is common in Firmicutes (65) and responsible for encoding β-glucuronidase enzyme (32, 66), which in turn removes the glucuronic acid from conjugated substrates such as steroid hormones and other xenobiotics secreted to the intestinal lumen through the bile duct after being processed by hepatic glucuronidation to promote reabsorption of them as aglycone steroid hormones or xenobiotics via enterohepatic circulation again (67–69). This aligns with our PICRUST findings (**Figure 4D**) that predicted an increase amino acids and pyrimidine metabolism in OVX+END mice, as compared to the lipid metabolism predicted in OVX+VEH and the carbohydrate metabolism predicted in the Naïve group.

Several mechanisms have been attributed to the beneficial effects of microbiome in health maintenance including the production of short chain fatty acids (SCFAs) (70). Enrichments of *Ruminococcus gnavus* in Naïve and Naïve+END groups (**Figure 4C**) and Lachnospiraceae in Naïve+END groups are all major producers of short chain fatty acids (SCFAs) and may explain the elevated levels of propionic (**Figure 5C**) and Iso-butyric (**Figure 5E**) acids in the colonic flushes of respective groups (32, 70). However, butyric acid, which is the most beneficial SCFA for intestinal homeostasis, energy metabolism, and anti-inflammatory effects (71–74) was significantly higher in the Naïve group in comparison with all other groups (**Figure 5D**), which could be due to the abundance of *Ruminococcus gnavus* (**Figure 4C**). These data also suggested that decreased butyric acid production in Naïve+END and OVX+END groups may be related to increased inflammation seen in these groups.

Another major role of the microbiome is to metabolize estrogen hormone under the regulation of *gus* gene (32, 66). Any deviation in the steroid hormone production in the host circulation could lead to initiation of disease through microbiome dysregulation (16, 20, 22, 31, 57). Nevertheless, the balance of steroid production during the sexual cycle is necessary for reproduction (22). Long-term changes in reproductive hormones lead to shifts in gut microbiome (28–30) and the increase in progesterone levels during pregnancy is associated with an increase in beneficial bacteria like Bifidobacterium (29). Gut dysbiosis certainly influences immune system function (41, 52, 75) as well as reproductive physiology through the modulation of reproductive hormones function and vice versa (22, 28, 29, 57, 66). In our study, we found through metabolic evaluation of the immune cell in the peritoneum of endometriotic and non-endometriotic mice (**Figures 6**, **7**) that the immunometabolism of these cells shifted, likely due to the microbiota and estrobolome dysbiosis (20, 22, 58). Estrogen plays important regulatory role in the maintenance of the immune system functions and to counteract inflammations and oxidative stress, thus, the reproductive system-gut axis and its related metabolism and metabolites are important regulators to stabilize the health and homeostasis (9, 76).

In summary, the current study suggests that reversing the endometriotic gut microbiota by, for example, through fecal transplants from healthy donors or increasing diet enrichment with prebiotics and/or probiotics could help in suppressing the endometriosis pathogenesis as well as severity which may result in minimizing its driven complications.

## Data availability statement

The datasets presented in this study can be found in online repositories, https://www.ncbi.nlm.nih.gov/geo, under accession number GSE248213.

## Ethics statement

The animal study was approved by Institutional Animal Care and Use Committee (IUCAC) in the University of South Carolina before performance of any study experiments (AUP2374). The study was conducted in accordance with the local legislation and institutional requirements.

## Author contributions

HA: Conceptualization, Data curation, Formal analysis, Methodology, Software, Validation, Visualization, Writing – original draft, Writing – review & editing. AM: Data curation, Formal analysis, Methodology, Writing – review & editing. NS: Formal analysis, Methodology, Writing – review & editing. RB: Writing – review & editing. IC: Formal analysis and Methodology. MN: Conceptualization, Funding acquisition, Project administration, Resources, Supervision, Writing – review & editing. PN: Conceptualization, Funding acquisition, Project administration, Resources, Supervision, Writing – review & editing.
