# Supplementary material for: Estrobolome dysregulation is associated with altered immunometabolism in a mouse model of endometriosis
Source: Front Endocrinol (Lausanne). 2023 Dec 8;14:1261781. doi: 10.3389/fendo.2023.1261781 (PMC10748389; doi:10.3389/fendo.2023.1261781)
Supplement: Supplementary file 1 [file DataSheet_1.pdf]

Table S1: Statistical analysis of data presented in Figure 2 with p values.						
PANEL	Naïve vs Naïve+END	Naïve vs OVX+VEH	Naïve vs OVX+END	Naïve+END vs OVX+VEH	Naïve+END vs OVX+END	OVX+VEH vs OVX+END
C	>0.9999	0.8434	**0.0016**	0.8578	**0.0013**	**0.0004**

Each cell shows p values and those with bold digits show statistically significant differences.

Table S2: Statistical analysis of data with p values presented in Figure 3.							
PANEL		Naïve vs Naïve+END	Naïve vs OVX+VEH	Naïve vs OVX+END	Naïve+END vs OVX+VEH	Naïve+END vs OVX+END	OVX+VEH vs OVX+END
A	left	**0.0013**	0.9956	**0.0197**	**0.0026**	0.6299	**0.0399**
	right	>0.9999	0.9574	0.834	0.9601	0.8395	0.9734
B		0.2527	0.9288	**0.001**	0.6654	**0.0577**	**0.0043**
D		0.1935	>0.9999	**0.0311**	0.2253	0.8401	**0.0373**
E		>0.9999	0.9629	**0.0418**	0.9518	**0.046**	**0.0119**
F		**0.0025**	**0.0139**	**0.0006**	0.8938	0.9334	0.4776
G		**0.0024**	0.072	**0.0008**	0.4224	0.9788	0.1875
H		**<0.0001**	**0.0001**	**<0.0001**	**0.0051**	0.999	**0.0067**

Each cell shows p values and those with bold digits show statistically significant differences.

Table S3: Statistical analysis of data with p values presented in Figure 4.							
PANEL C microbiota		Naïve vs Naïve+END	Naïve vs OVX+VEH	Naïve vs OVX+END	Naïve+END vs OVX+VEH	Naïve+END vs OVX+END	OVX+VEH vs OVX+END
C	Tenericutes phylum	**<0.0001**	**<0.0001**	**<0.0001**	**<0.0001**	0.2193	**<0.0001**
	Molicutes class	**<0.0001**	**<0.0001**	**<0.0001**	**<0.0001**	0.2193	**<0.0001**
	Anaeroplasmatales order	**<0.0001**	**<0.0001**	**<0.0001**	**<0.0001**	0.2193	**<0.0001**
	Anaeroplasma genus	**<0.0001**	**<0.0001**	**<0.0001**	**<0.0001**	0.2193	**<0.0001**
	Chlostrediales order	0.5077	**0.0259**	0.9451	0.2705	0.25	**0.0099**
	Coprococcus genus	**0.0237**	0.5461	>0.9999	**0.0023**	**0.0237**	0.5461
	Dehalobacterium genus	0.9319	0.3276	0.3276	0.6452	0.135	**0.0177**
	Ruminococcus gnavus	0.7919	**0.0002**	**0.0028**	**<0.0001**	**0.0006**	0.298

Each cell shows p values and those with bold digits show statistically significant differences.

Table S4: Statistical analysis of data with p values presented in Figure 5.						
Panel	Naïve vs Naïve+END	Naïve vs OVX+VEH	Naïve vs OVX+END	Naïve+END vs OVX+VEH	Naïve+END vs OVX+END	OVX+VEH vs OVX+END
B	**<0.0001**	**0.0003**	**0.0447**	**<0.0001**	**<0.0001**	**<0.0001**
C	**<0.0001**	**<0.0001**	**<0.0001**	**<0.0001**	**0.0002**	0.3376
D	**<0.0001**	**<0.0001**	**<0.0001**	**<0.0001**	**0.0001**	**<0.0001**
E	0.6821	**<0.0001**	**<0.0001**	**<0.0001**	**<0.0001**	0.4989
F	**<0.0001**	**0.0002**	**<0.0001**	**<0.0001**	**<0.0001**	**0.0003**
G	0.4835	**<0.0001**	**<0.0001**	**<0.0001**	**<0.0001**	0.9191

Each cell shows p values and those with bold digits show statistically significant differences.
